# COVID-19 as a Natural Disaster: Focusing on Exposure and Vulnerability for Response

**DOI:** 10.1017/dmp.2020.279

**Published:** 2020-07-27

**Authors:** Hamed Seddighi

**Affiliations:** Health and Social Welfare, Student Research Committee, University of Social Welfare and Rehabilitation Sciences, Tehran, Iran

**Keywords:** COVID-19, exposure, inequality, vulnerability

The coronavirus disease (COVID-19) pandemic is described as humanity’s worst crisis since World War II.^1^ The social, political, and financial consequences of this pandemic will remain for years and decades.^2,3^ Impacts of COVID-19 are not distributed equally. In a study in New York City, it is shown that moving from the poorest zip codes to the richest zip codes is associated with an increase in the fraction of negative COVID-19 test results from 38 to 65%.^4^ The World Bank discussed in a report that COVID-19 affects men and women differently and proposed to greater gender equality in related policies.^5^ On the other hand, the COVID-19 consequences will raise vulnerability.^6^ Faheem et al. estimated that for each percentage point reduction in the global economy, more than 10 million people are plunged into poverty worldwide.^7^

According to the International Federation of Red Cross and Red Crescent Societies, pandemics are classified as a natural hazard. Disaster risk has a relationship with the type of disaster, vulnerability, and exposure or as a formula (risk = disaster*vulnerability*exposure). For reducing risks, beside the disaster prevention, it is necessary to reduce vulnerability and exposure. These 2 elements have a social dimension. Scholars in social vulnerability discussing hazard risks is a reflection of socially constructed vulnerability. Social vulnerabilities intersect, interact, overlap, and cluster together in their hazards’ impacts.^8^ Social vulnerabilities are determinants, such as age, gender, disability, race, ethnicity, caste, tribe, religion, class, status, education, occupation, income, and residence.^9^ On the other hand, exposure is a social construction also. Lifestyle choices are affected by life’s chances that are defined by the environment in which people live.^10^ People with a social vulnerability as a result of socio-historical and economic saturation are living in hazardous areas with poor housing, that is, exposing themselves to further risks^11^ – thus, the hazard risk shaped by social vulnerability and social exposure. As a result, if the governments and non-governmental organizations decided to design an intervention, the entry point should be reducing vulnerabilities of individuals toward COVID-19.

Wrong decisions and neglecting social exposure and social vulnerability in COVID-19 response will have negative impacts in the future. Many times, the best effort to solve a problem can literally make it worse. Frequently, well-intentioned solutions for problems generate policy resistance. Such phenomena are called the *counterintuitive behavior of social systems*. Policy resistance appears because the full extent of feedbacks operating in the system is not understood. In complex systems, such as a country, the cause (policy) and effect (consequences) are often distant in time and space. Thus, in the case of COVID-19 interventions, the government with a policy intended to solve the problems of the country. However, the fastest solution is not the best one, as Sir Thomas More, the English lawyer, social philosopher, and author (1516) would seem to agree in his own words, “And it will fall out as in a complication of the disease, that by applying a remedy to one sore, you will provoke another; and that which removes the … one ill symptom produces others.”^12^
